# The platelet to lymphocyte ratio is a potential inflammatory marker predicting the effects of adjuvant chemotherapy in patients with stage II colorectal cancer

**DOI:** 10.1186/s12885-021-08521-0

**Published:** 2021-07-08

**Authors:** Yu Fu, Xiaowan Chen, Yongxi Song, Xuanzhang Huang, Quan Chen, Xinger Lv, Peng Gao, Zhenning Wang

**Affiliations:** Department of Surgical Oncology and General Surgery, The First Hospital of China Medical University; Key Laboratory of Precision Diagnosis and Treatment of Gastrointestinal Tumors (China Medical University), Ministry of Education, 155 North Nanjing Street, Heping District, Shenyang, 110001 China

**Keywords:** Colorectal cancer, Stage II, Adjuvant chemotherapy, Inflammatory marker, Platelet to lymphocyte ratio

## Abstract

**Background:**

The effects of adjuvant chemotherapy in patients with stage II colorectal cancer (CRC) has been in controversy for a long time. Our study aimed to find an effective inflammatory marker to predict the effects of chemotherapy.

**Methods:**

Seven hundred eight stage II CRC patients in our institution were included. The subpopulation treatment effect pattern plot (STEPP) analysis was used to determine the optimal inflammatory marker and cut-off value. Propensity score matching (PSM) was performed to balance discrepancy between the chemotherapy and non-chemotherapy group. Survival analyses based on overall survival (OS) and cancer-specific survival (CSS) were performed with Kaplan-Meier methods with log-rank test and Cox proportional hazards regression. The restricted mean survival time (RMST) was used to measure treatment effect.

**Results:**

The platelet to lymphocyte ratio (PLR) was chosen as the optimal marker with a cut-off value of 130 according to STEPP. In OS analysis, PLR was significantly associated with the effects of chemotherapy (interaction *p* = 0.027). In the low-PLR subgroup, the chemotherapy patients did not have a longer OS than the non-chemotherapy patients (HR: 0.983, 95% CI: 0.528–1.829). In the high-PLR subgroup, the chemotherapy patients had a significantly longer OS than the non-chemotherapy patients (HR: 0.371, 95% CI: 0.212–0.649). After PSM, PLR was still associated with the effects of chemotherapy. In CSS analysis, PLR was not significantly associated with the effects of chemotherapy (interaction *p* = 0.116). In the low-PLR subgroup, the chemotherapy patients did not have a longer CSS than the non-chemotherapy patients (HR: 1.016, 95% CI: 0.494–2.087). In the high-PLR subgroup, the chemotherapy patients had a longer CSS than the non-chemotherapy patients (HR: 0.371, 95% CI: 0.212–0.649). After PSM, PLR was not associated with the effects of chemotherapy.

**Conclusions:**

PLR is an effective marker to predict the effects of chemotherapy in patients with stage II CRC.

**Supplementary Information:**

The online version contains supplementary material available at 10.1186/s12885-021-08521-0.

## Background

Colorectal cancer (CRC) is the third most commonly diagnosed cancer and the second most lethal [1]. Radical resection is the principal curative treatment for patients with nonmetastatic CRC. Adjuvant chemotherapy is a crucial means to improve additional survival benefits beyond those from surgery. Remarkably, the effects of adjuvant chemotherapy in patients with CRC is highly likely to be stage-specific. For patients with stage III CRC, adjuvant chemotherapy has been demonstrated to greatly improve survival [2, 3]. However, survival benefits from chemotherapy in stage II CRC are still unclear. Many studies tried to find survival benefits from chemotherapy in stage II CRC but failed. In the MOSAIC study and another study based on the ACCENT database, oxaliplatin-based chemotherapy was not found to increase survival in the first 6 to 10 years after surgery [4, 5]. In the QUASAR study, the absolute improvement in survival after chemotherapy with fluorouracil was only 3.6% [6]. However, one study has demonstrated that treatment with 5-FU/LV after surgery confers significant survival benefits beyond those of surgery alone in stage II CRC [7]. On the basis of these and other studies, both the NCCN and ASCO guidelines suggest offering chemotherapy to patients with high-risk stage II CRC [8–12]. Patients are considered high-risk if they have T4 depth of invasion, peritumoral lymphatic/venous invasion, a histological grade of 3 or greater, R1-R2 margin status, bowel obstruction or perforation, elevated carcinoembryonic antigen (CEA) exceeding 5 ng/mL or fewer than 12 nodes retrieved. However, these high-risk characteristics are determined on the basis of prognosis instead of the additional survival benefit from chemotherapy. The guidelines also indicated that no available data correlated risk features and selection of chemotherapy in high-risk stage II patients [13].

In the search for characteristics predicting the effects of chemotherapy on CRC and other cancers, many inflammatory markers have been used [14–16]. Inflammatory markers including the platelet to lymphocyte ratio (PLR), neutrophil to lymphocyte ratio (NLR), lymphocyte to monocyte ratio (LMR), prognostic nutritional index (PNI) and others have been shown to be strongly associated with not only cancer prognosis [17–21], but also with chemo-resistance and chemotherapeutic responses [22–26]. Nevertheless, previous studies of inflammatory markers have focused on advanced cancers which are usually accompanied by metastasis. However, no such studies have been performed on stage II CRC.

In this study, we aimed to investigate the ability of inflammatory markers to predict the effects of chemotherapy in patients with stage II CRC and to identify an effective method allowing clinicians to distinguish the population in which chemotherapy is effective.

## Methods

### Participants and criteria

We retrospectively analyzed patients with stage II CRC who received surgical treatment at the Department of Surgical Oncology and General Surgery, First Affiliated Hospital of China Medical University between August 2010 and August 2015. All patients enrolled in this study had undergone radical resection (R0) for the first time. Furthermore, a portion of the patients had received adjuvant chemotherapy as a single-agent therapy regimen with fluoropyrimidines or as a combination therapy regimen with fluoropyrimidines and oxaliplatin. Patients with high-risk factors were proposed to receive chemotherapy. For patients without high-risk factors, chemotherapy reception was decided according to the will of themselves and their relatives. The single-agent therapy was comprised one of the following regimens. (1) Day 1: leucovorin 400 mg/m^2^ intravenous injection (IV), followed by a 400 mg/m^2^ IV bolus of 5-FU, followed by 2400 mg/m^2^ as a 46–48 h continuous infusion. The cycle was repeated every 2 weeks for 6 months of perioperative therapy. (2) Days 1–14: capecitabine 1000–1250 mg/m^2^ orally twice daily. The cycle was repeated every 3 weeks for 6 months of perioperative therapy. The combination therapy comprised one of the following regimens. (1) Day 1: oxaliplatin 85 mg/m^2^ IV over 2 h + leucovorin 400 mg/m^2^ IV over 2 h, followed a 400 mg/m^2^ IV bolus of 5-FU, followed by 2400 mg/m^2^ as a 46–48 h continuous infusion. The cycle was repeated every 2 weeks for 6 months of perioperative therapy. (2) Day 1: oxaliplatin 130 mg/m^2^ IV. Days 1–14: capecitabine 1000 mg/m^2^ orally twice daily. The cycle was repeated every 3 weeks for 6 months of perioperative therapy. All patients were age 18 years or older and had provided signed informed consent and agreed to the use of their personal data for research. The primary inclusion criteria were as follows: (1) adenocarcinoma of colon or rectum diagnosed by histopathology, (2) stage II classified on the basis of the eighth edition of the AJCC/UICC TNM classification system, (3) complete patient information available, including baseline characteristics, follow-up and laboratory data. (4) all blood samples obtained within 1 week before the operation. Patients were excluded from this study on the basis of the following criteria: (1) neoadjuvant chemotherapy, (2) long-term use of anti-coagulant or anti-inflammatory medicine before surgery, (3) surgery in emergency circumstances, including obstruction and perforation. Follow-up was completed for all patients until December 2018. The median follow-up period was 49 months. The clinicopathological characteristics, including chemotherapy status, sex, age, tumor localization, tumor size, differentiation, T category, numbers of examined lymph nodes and status of vessel carcinoma embolus and cerebrovascular and cardiovascular diseases (CCVD), and laboratory data were collected from the electronic medical records. The neutrophil, platelet and lymphocyte counts were collected with routine blood tests. Albumin levels were determined with the hepatic function tests. PLR was defined as the absolute platelet count divided by the absolute lymphocyte count. NLR was defined as the absolute neutrophil count divided by the absolute lymphocyte count. LMR was defined as the absolute lymphocyte count divided by the absolute monocyte count. PNI was defined as 10 × albumin level (g/dl) + 0.005 × lymphocyte count (per mm3) [27].

### Statistical analysis

Subpopulation Treatment Effect Pattern Plot (STEPP) analysis was used to determine the optimal predictive inflammatory markers and cut-off values [28, 29]. The Mann-Whitney U test and chi-square test were used to compare the differences in the inflammatory markers and other characteristics, respectively. Our survival analysis was based on overall survival (OS) and cancer-specific survival (CSS). The Kaplan-Meier method with the log-rank test was used to compare survival differences between the chemotherapy and non-chemotherapy groups. Univariate Cox proportional hazard regression analysis was used to calculate the hazard ratio (HR). Multivariate Cox proportional hazard regression analysis was used to assess the interactions between the clinicopathological characteristics and the effect of chemotherapy.

In a survival analysis, calculation of the survival probability at a specific time point is difficult. The restricted mean survival time (RMST) can be used as an alternative to summarize the profile [30, 31]. The RMST was defined as the mean survival time by a certain time point with clinical meaning in the period of a study. It was equal to the area under the survival curve until the selected time point. The difference in RMST can be explained as the increase or decrease in survival time caused by a certain treatment. Consequently, the RMST was considered as an effective method to measure treatment effects. In our study, the RMST at 60 months, calculated by Kaplan-Meier survival analysis, was used.

In an observational study, researchers cannot control the treatment distribution, and large differences may exist in the covariates between the treated and non-treated groups. These differences are highly likely to cause a bias in treatment effect estimates. In our study, propensity score matching (PSM) [32–34] was performed to balance the covariates in the two groups and decrease this bias.

Statistical evaluation was performed using SPSS software version 25.0 (IBM Corporation, Armonk, NY, USA) and R software version 3.6.1 (St. Louis, Missouri, USA). All statistical tests were two-sided, and a *P*-value of less than 0.05 was considered to indicate a statistically significant difference.

## Results

### Patient characteristics

After the selection, a total of 708 patients with stage II CRC were included in the study. The clinicopathological characteristics of patients were summarized and compared with the chi-square test in Table 1 and Mann-Whitney U test in Table 2. A total of 447 (63.1%) patients received chemotherapy and 261 (36.9%) patients did not; 431 (60.9%) patients were men and 277 (39.1%) were women; 354 (50.0%) patients had colon cancer and 354 (50.0%) patients had rectal cancer. The median age of the patients was 63 years (range 23–88 years). According to the chi-square test and Mann-Whitney U test, significant differences in age, differentiation, T category, cerebrovascular and cardiovascular diseases, LMR level and PNI level existed between chemotherapy and non-chemotherapy patients. Higher proportions of advanced age (46.7% vs. 11.0%, *p* < 0.001), poor differentiation (9.6% vs. 6.0%, *p* = 0.038) and positive cardio-cerebrovascular comorbidities (16.9% vs. 10.7%, *p* = 0.019) were observed in the non-chemotherapy patients, whereas the chemotherapy patients were more often diagnosed with the T4 category (67.3% vs. 54.0%, p < 0.001).

**Table 1:** Characteristics of patients

Characteristics		Total	Non-Chemotherapy	Chemotherapy	*P* value^a^
Sex	Male	431 (60.9%)	156 (59.8%)	275 (61.5%)	0.645
	Female	277 (39.1%)	105 (40.2%)	172 (38.5%)	
Age (years)	≤55	163 (23.0%)	32 (12.3%)	131 (29.3%)	< 0.001
	56–60	125 (17.7%)	23 (8.8%)	102 (22.8%)	
	61–65	146 (20.6%)	40 (15.3%)	106 (23.7%)	
	66–70	103 (14.5%)	44 (16.9%)	59 (13.2%)	
	> 70	171 (24.2%)	122 (46.7%)	49 (11.0%)	
Location	Rectum	354 (50.0%)	119 (45.6%)	235 (52.6%)	0.073
	Colon	354 (50.0%)	142 (54.4%)	212 (47.4%)	
Size (cm)	≤5.0	395 (55.8%)	144 (55.2%)	251 (56.2%)	0.954
	> 5.0	303 (42.8%)	113 (43.3%)	190 (42.5%)	
	Unknown	10 (1.4%)	4 (1.5%)	6 (1.3%)	
Differentiation	Well-moderate	654 (92.4%)	234 (89.7%)	420 (94.0%)	0.038
	Poor	52 (7.3%)	25 (9.6%)	27 (6.0%)	
	Unknown	2 (0.3%)	2 (0.8%)	0 (0)	
T category	3	266 (37.6%)	120 (46.0%)	146 (32.7%)	< 0.001
	4	442 (62.4%)	141 (54.0%)	301 (67.3%)	
CEA^b^ (ng/mL)	< 5	388 (54.8%)	135 (51.7%)	253 (56.6%)	0.058
	≥5	237 (33.5%)	101 (38.7%)	136 (30.4%)	
	Unknown	83 (11.7%)	25 (9.6%)	58 (13.0%)	
Examined lymph nodes	< 12	186 (26.3%)	75 (28.7%)	111 (24.8%)	0.255
	≥12	522 (73.7%)	186 (71.3%)	336 (75.2%)	
Vessel carcinoma embolus	Negative	683 (96.5%)	251 (96.2%)	432 (96.6%)	0.741
	Positive	25 (3.5%)	10 (3.8%)	15 (3.4%)	
CCVD^c^	Negative	616 (87.0%)	217 (83.1%)	399 (89.3%)	0.019
	Positive	92 (13.0%)	44 (16.9%)	48 (10.7%)	
PLR^d^	≤130	371 (52.4%)	134 (51.3%)	237 (53.0%)	0.666
	> 130	337 (47.6%)	127 (48.7%)	210 (47.0%)	

^a^ P value of the Chi-square test

^b^ CEA: carcinoembryonic antigen

^c^ CCVD: cerebrovascular and cardiovascular diseases

^d^ PLR: platelet to lymphocyte ratio

**Table 2:** Inflammatory markers of patients

Marker	Total		Non-chemotherapy		Chemotherapy		P value^a^
	Median	Interquartile Range	Median	Interquartile Range	Median	Interquartile Range	
PLR^b^	127.60	71.64	128.49	74.81	125.84	69.56	0.562
NLR^c^	1.94	1.28	2.02	1.27	1.88	1.30	0.080
LMR^d^	4.21	2.58	3.91	2.53	4.28	2.56	0.008
PNI^e^	50.28	7.33	48.90	8.05	50.75	6.75	< 0.001

^a^ P value of Mann-Whitney U test

^b^ PLR: platelet to lymphocyte ratio

^c^ NLR: neutrophil to lymphocyte ratio

^d^ LMR: lymphocyte to monocyte ratio

^e^ PNI: prognostic nutritional index

### Optimal inflammatory marker

STEPP was performed by plotting the level of the inflammatory markers NLR, PLR, LMR and PNI on the x-axis and the cumulative mortality at 60 months, as measured by the Kaplan-Meier method, on the y-axis to compare the OS between the chemotherapy and non-chemotherapy patients in different subgroups divided by the levels of the inflammatory markers.

**Fig. 1:**
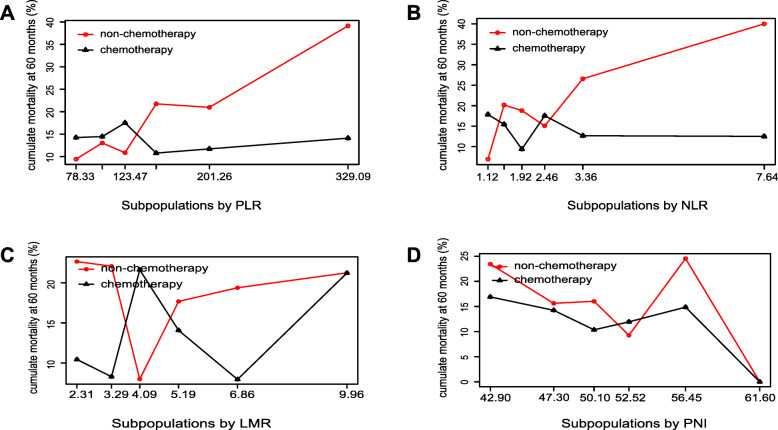
STEPP analysis of the concerned inflammatory markers. (**A**) The result of PLR. (**B**) The result of NLR. (**C**) The result of LMR. (**D**) The result of PNI

In the PLR-related analysis, when the PLR was > 130, the cumulative mortality of the non-chemotherapy patients was significantly and continually higher than that of the chemotherapy patients, whereas the tendency was just in contrary when the PLR was < 130 (Fig. 1A). Furthermore, no such tendency was found in the analyses of the other inflammatory markers (Fig. 1B, C and D). These results indicated that the PLR levels were closely associated with the survival benefits of chemotherapy. We further used a PLR level of 130 as the cut-off value to distinguish population in which chemotherapy was effective. We divided the patients into a high-PLR subgroup (PLR ≥130) and low-PLR subgroup (PLR < 130) and performed chi-square analysis to compare PLR levels between the chemotherapy and non-chemotherapy patients (Table 1).

### Overall survival analysis

**Fig. 2:**
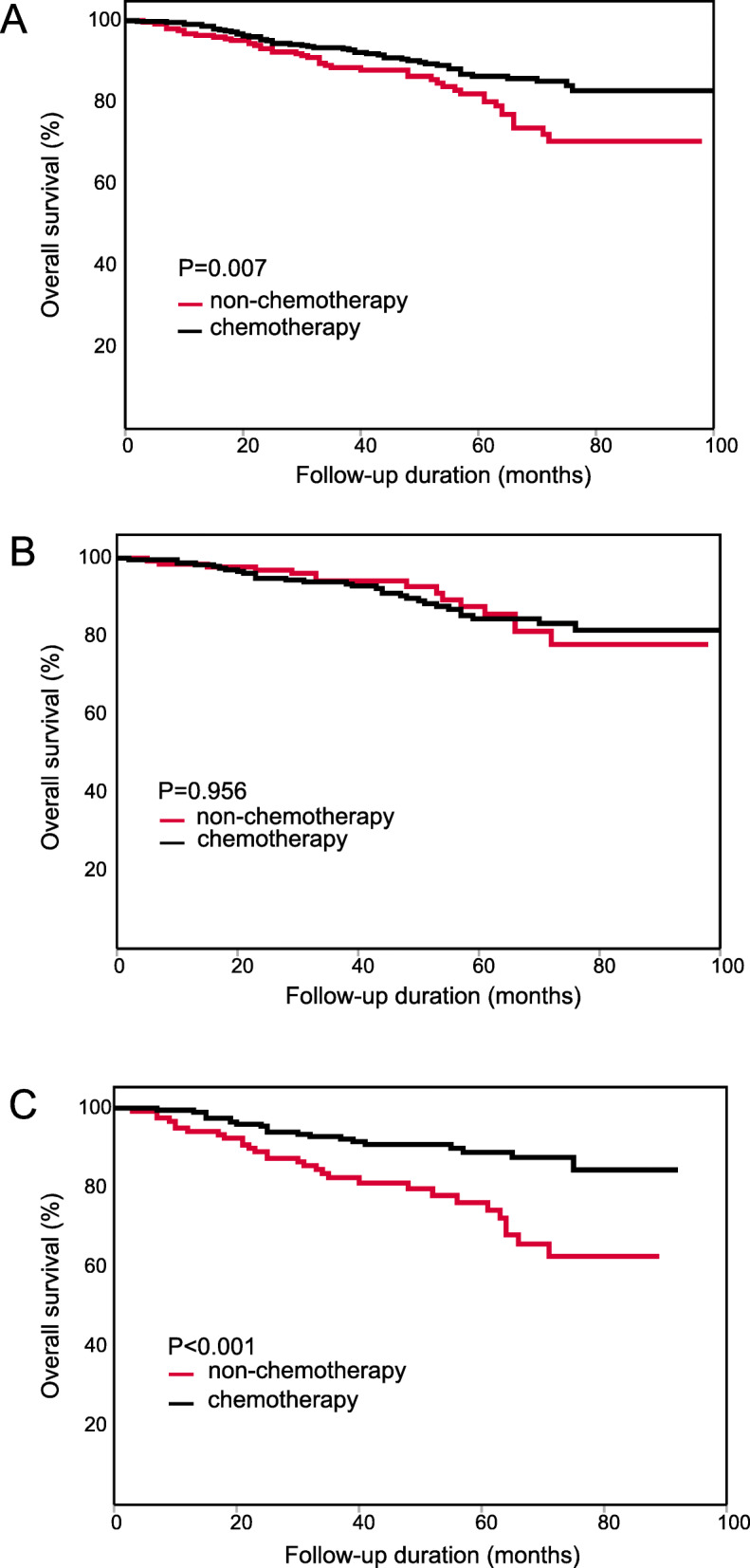
Kaplan-Meier OS curve of patients with stage II CRC. (**A**) The result of all patients. (**B**) The result of the low-PLR subgroup. (**C**) The result of the high-PLR subgroup

For all 708 patients with stage II CRC, chemotherapy patients had a longer OS than non-chemotherapy patients (HR: 0.580, 95%CI: 0.388–0.866, Fig. 2A). We also divided the patients into subgroups according to several characteristics including PLR level. The results of subgroup survival analysis indicated that the chemotherapy patients with the following characteristics had a longer OS than the non-chemotherapy patients: male (HR: 0.592, 95%CI: 0.360–0.971), rectal cancer (HR: 0.539, 95% CI: 0.323–0.900), well or moderate differentiation (HR: 0.569, 95% CI: 0.373–0.868), T4 category (HR: 0.501, 95% CI: 0.318–0.791), number of examined lymph nodes≥12 (HR: 0.500, 95% CI: 0.300–0.835), and PLR level > 130 (HR: 0.371, 95% CI: 0.212–0.649). The detailed results are shown in Table 3.

**Table 3:** OS analysis for all patients

Characteristics		RMST^b^ (95%CI)		HR^c^ (95%CI)	P value^a^	Interaction p
		Non-chemotherapy	Chemotherapy			
Total	–	53.7 (51.9–55.2)	56.2 (55.2–57.0)	0.580 (0.388–0.866)	0.007	–
Sex	Male	53.2 (50.7–55.1)	55.9 (54.4–57.0)	0.592 (0.360–0.971)	0.035	0.751
	Female	54.5 (51.7–56.4)	56.8 (54.8–58.0)	0.560 (0.282–1.112)	0.093	
Age (years)	≤55	54.3 (48.0–57.6)	56.3 (53.8–57.8)	0.620 (0.225–1.708)	0.350	0.844
	56–60	55.4 (47.4–58.5)	56.8 (54.4–58.2)	0.725 (0.199–2.634)	0.623	
	61–65	53.3 (46.5–56.9)	55.7 (52.9–57.5)	0.636 (0.240–1.683)	0.357	
	66–70	53.2 (48.1–56.4)	57.3 (53.6–58.8)	0.355 (0.118–1.063)	0.053	
	> 70	53.6 (50.9–55.7)	54.6 (49.5–57.1)	0.834 (0.371–1.875)	0.660	
Location	Rectum	52.2 (49.2–54.5)	55.6 (53.9–56.9)	0.539 (0.323–0.900)	0.016	0.479
	Colon	55.2 (52.8–56.9)	56.9 (55.4–58.1)	0.612 (0.321–1.168)	0.132	
Size (cm)	≤5.0	54.0 (51.2–55.8)	56.1 (54.5–57.2)	0.621 (0.361–1.067)	0.081	0.255
	> 5.0	53.7 (50.5–55.8)	56.3 (54.5–57.5)	0.561 (0.306–1.028)	0.058	
Differentiation	Well-moderate	53.9 (52.0–55.4)	56.3 (55.2–57.2)	0.569 (0.373–0.868)	0.008	0.396
	Poor	52.0 (43.2–56.6)	54.7 (46.8–57.8)	0.662 (0.177–2.471)	0.534	
T category	3	56.4 (53.7–58.0)	57.6 (55.5–58.6)	0.654 (0.277–1.543)	0.328	0.382
	4	51.8 (49.2–54.0)	55.6 (54.2–56.8)	0.501 (0.318–0.791)	0.002	
CEA^d^ (ng/mL)	< 5	54.6 (51.9–56.4)	56.7 (55.3–57.8)	0.585 (0.323–1.060)	0.073	0.482
	≥5	53.5 (50.1–55.7)	55.1 (52.8–56.8)	0.734 (0.384–1.404)	0.348	
Examined lymph nodes	< 12	52.0 (47.5–54.9)	53.6 (50.2–55.6)	0.793 (0.414–1.520)	0.483	0.415
	≥12	54.5 (52.3–56.0)	57.1 (55.9–57.9)	0.500 (0.300–0.835)	0.007	
PLR^e^	≤130	56.0 (53.6–57.6)	56.0 (54.3–57.2)	0.983 (0.528–1.829)	0.956	0.027
	> 130	51.3 (47.8–53.7)	56.5 (54.8–57.7)	0.371 (0.212–0.649)	< 0.001	

^a^ P value of the log-rank test

^b^ RMST: the restricted mean survival time

^c^ HR: Hazard Ratio, chemotherapy patients vs. non-chemotherapy patients

^d^ CEA: carcinoembryonic antigen

^e^ PLR: platelet to lymphocyte ratio

According to multivariate Cox survival analysis, PLR was the only characteristic significantly associated with the effects of chemotherapy (interaction *p* = 0.027). In the low-PLR subgroup, the chemotherapy patients did not obtain OS benefits beyond those of the non-chemotherapy patients (HR: 0.983, 95% CI: 0.528–1.829, Fig. 2B). However, in the high-PLR subgroup, the chemotherapy patients had a significantly longer OS than the non-chemotherapy patients (HR: 0.371, 95% CI: 0.212–0.649, Fig. 2C). These results showed that PLR could distinguish the population in which chemotherapy is effective.

**Fig. 3:**
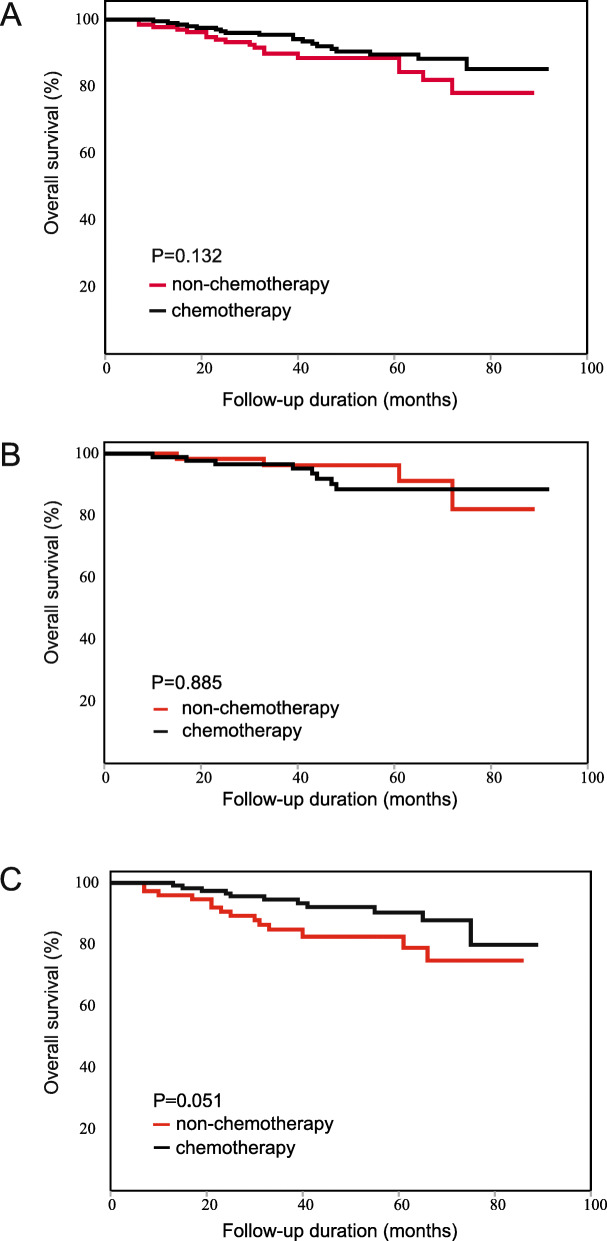
Kaplan-Meier OS curve of patients with stage II colon cancer. (**A**) The result of all patients. (**B**) The result of the low-PLR subgroup. (**C**) The result of the high-PLR subgroup

**Fig. 4:**
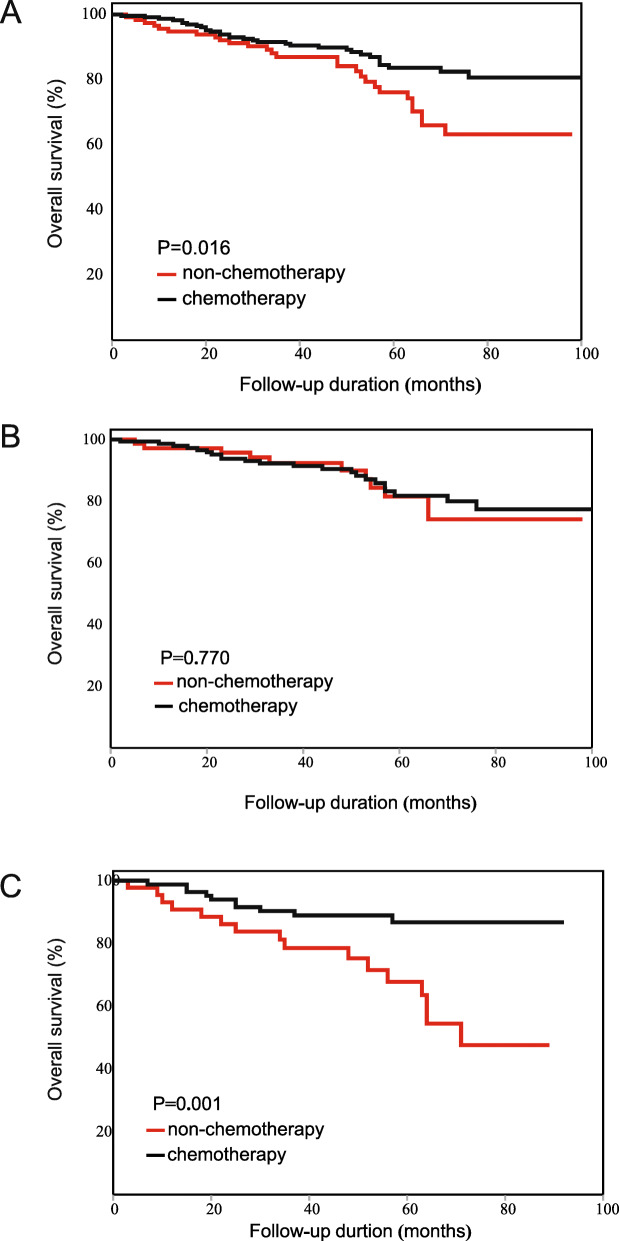
Kaplan-Meier OS curve of patients with stage II rectal cancer. (**A**) The result of all patients. (**B**) The result of the low-PLR subgroup. (**C**) The result of the high-PLR subgroup

Considering that the high-risk factors and standard therapeutic approaches in stage II colon cancer and rectal cancer were not completely consistent, we also performed survival analyses in the colon cancer subgroup and the rectal cancer subgroup separately. The results of both subgroups were in accordance with the results for all patients with CRC, PLR was significantly associated with the effects of chemotherapy in both subgroups. For colon cancer: the chemotherapy patients did not have a longer OS than the non-chemotherapy patients (HR: 0.612, 95%CI: 0.321–1.168, Fig. 3A). In low-PLR subgroup, the chemotherapy patients did not have a longer OS (HR: 1.093, 95%CI: 0.328–3.647, Fig. 3B). In high-PLR subgroup, the chemotherapy patients had a longer OS than non-chemotherapy patients (HR: 0.464, 95% CI: 0.211–1.024, Fig. 3C). For rectal cancer: the chemotherapy patients had a longer OS than the non-chemotherapy patients (HR: 0.539, 95%CI: 0.323–0.900, Fig. 4A). In low-PLR subgroup, the chemotherapy patients did not have a longer OS (HR: 0.898, 95%CI: 0.435–1.854, Fig. 4B). In high-PLR subgroup, the chemotherapy patients had a significantly longer OS (HR: 0.289, 95% CI: 0.131–0.638, Fig. 4C). The detailed results of subgroup analysis are shown in Tables 4 and 5.

**Table 4:** OS analysis for patients with colon cancer

Characteristics		RMST^b^ (95%CI)		HR^c^ (95%CI)	P value^a^	Interaction p
		Non-chemotherapy	Chemotherapy			
Total	–	55.2 (52.8–57.0)	56.9 (55.2–58.0)	0.612 (0.321–1.168)	0.132	–
Sex	Male	55.0 (51.1–57.2)	56.7 (54.3–58.2)	0.636 (0.269–1.502)	0.298	0.887
	Female	55.4 (51.1–57.7)	57.2 (54.5–58.6)	0.599 (0.225–1.599)	0.301	
Age (years)	≤55	52.0 (40.0–57.2)	57.5 (54.0–58.9)	0.262 (0.062–1.107)	0.050	0.179
	56–60	56.0 (38.7–59.5)	57.0 (52.2–58.9)	0.849 (0.095–7.617)	0.884	
	61–65	55.4 (43.1–58.8)	55.7 (51.1–57.8)	0.882 (0.176–4.421)	0.879	
	66–70	52.9 (45.9–56.5)	–	0.015 (0.001–10.71)	0.014	
	> 70	56.8 (53.0–58.5)	54.9 (47.8–58.0)	1.569 (0.442–5.569)	0.482	
Size (cm)	≤5.0	55.6 (51.7–57.6)	56.3 (53.7–57.9)	0.849 (0.341–2.114)	0.724	0.254
	> 5.0	53.7 (50.5–55.8)	56.3 (54.5–57.5)	0.452 (0.178–1.146)	0.086	
Differentiation	Well-moderate	53.9 (52.0–55.4)	56.3 (55.2–57.2)	0.621 (0.310–1.246)	0.175	0.990
	Poor	52.0 (43.2–56.6)	54.7 (46.8–57.8)	0.594 (0.099–3.561)	0.564	
T category	3	56.4 (53.7–58.0)	57.6 (55.5–58.6)	0.581 (0.116–2.907)	0.504	0.746
	4	51.8 (49.2–54.0)	55.6 (54.2–56.8)	0.575 (0.284–1.165)	0.119	
CEA^d^ (ng/mL)	< 5	54.6 (51.9–56.4)	56.7 (55.3–57.8)	0.606 (0.219–1.675)	0.329	0.522
	≥5	53.5 (50.1–55.7)	55.1 (52.8–56.8)	0.793 (0.305–2.061)	0.633	
Examined lymph nodes	< 12	52.0 (47.5–54.9)	53.6 (50.2–55.6)	0.626 (0.216–1.816)	0.383	0.949
	≥12	54.5 (52.3–56.0)	57.1 (55.9–57.9)	0.670 (0.293–1.534)	0.340	
PLR^e^	≤130	56.0 (53.6–57.6)	56.0 (54.3–57.2)	1.093 (0.328–3.647)	0.885	0.276
	> 130	51.3 (47.8–53.7)	56.5 (54.8–57.7)	0.464 (0.211–1.024)	0.051	

^a^ P value of the log-rank test

^b^ RMST: the restricted mean survival time

^c^ HR: Hazard Ratio, chemotherapy patients vs. non-chemotherapy patients

^d^ CEA: carcinoembryonic antigen

^e^ PLR: platelet to lymphocyte ratio

**Table 5:** OS analysis for patients with rectal cancer

Characteristics		RMST^b^ (95%CI)		HR^c^ (95%CI)	P value^a^	Interaction p
		Non-chemotherapy	Chemotherapy			
Total	–	53.7 (51.9–55.2)	56.2 (55.2–57.0)	0.539 (0.323–0.900)	0.016	–
Sex	Male	53.2 (50.7–55.1)	55.9 (54.4–57.0)	0.548 (0.298–1.006)	0.048	0.931
	Female	54.5 (51.7–56.4)	56.8 (54.8–58.0)	0.534 (0.205–1.391)	0.192	
Age (years)	≤55	54.3 (48.0–57.6)	56.3 (53.8–57.8)	1.204 (0.264–5.499)	0.810	0.314
	56–60	55.4 (47.4–58.5)	56.8 (54.4–58.2)	0.698 (0.141–3.459)	0.658	
	61–65	53.3 (46.5–56.9)	55.7 (52.9–57.5)	0.490 (0.142–1.694)	0.250	
	66–70	53.2 (48.1–56.4)	57.3 (53.6–58.8)	0.605 (0.143–2.551)	0.489	
	> 70	53.6 (50.9–55.7)	54.6 (49.5–57.1)	0.568 (0.190–1.701)	0.305	
Size (cm)	≤5.0	54.0 (51.2–55.8)	56.1 (54.5–57.2)	0.510 (0.260–1.002)	0.046	0.627
	> 5.0	53.7 (50.5–55.8)	56.3 (54.5–57.5)	0.631 (0.280–1.422)	0.261	
Differentiation	Well-moderate	53.9 (52.0–55.4)	56.3 (55.2–57.2)	0.519 (0.305–0.883)	0.014	0.309
	Poor	52.0 (43.2–56.6)	54.7 (46.8–57.8)	0.727 (0.102–5.208)	0.750	
T category	3	56.4 (53.7–58.0)	57.6 (55.5–58.6)	0.698 (0.253–1.925)	0.484	0.350
	4	51.8 (49.2–54.0)	55.6 (54.2–56.8)	0.431 (0.237–0.784)	0.004	
CEA^d^ (ng/mL)	< 5	54.6 (51.9–56.4)	56.7 (55.3–57.8)	0.568 (0.273–1.181)	0.124	0.523
	≥5	53.5 (50.1–55.7)	55.1 (52.8–56.8)	0.660 (0.273–1.598)	0.354	
Examined lymph nodes	< 12	52.0 (47.5–54.9)	53.6 (50.2–55.6)	0.885 (0.375–2.091)	0.780	0.169
	≥12	54.5 (52.3–56.0)	57.1 (55.9–57.9)	0.401 (0.208–0.771)	0.005	
PLR^e^	≤130	56.0 (53.6–57.6)	56.0 (54.3–57.2)	0.898 (0.435–1.854)	0.770	0.033
	> 130	51.3 (47.8–53.7)	56.5 (54.8–57.7)	0.289 (0.131–0.638)	0.001	

^a^ P value of the log-rank test

^b^ RMST: the restricted mean survival time

^c^ HR: Hazard Ratio, chemotherapy patients vs. non-chemotherapy patients

^d^ CEA: carcinoembryonic antigen

^e^ PLR: platelet to lymphocyte ratio

### Overall survival analysis after PSM

After PSM, 166 chemotherapy patients and 166 non-chemotherapy patients were paired. The characteristics of the matched patients are summarized and compared in Table 6. The differences in characteristics between chemotherapy and non-chemotherapy patients were acceptable.

**Table 6:** Characteristics of patients after PSM

Characteristics		Total	Non-chemotherapy	Chemotherapy	P value^a^
Sex	Male	199 (59.9%)	97 (58.4%)	102 (61.4%)	0.575
	Female	133 (40.1%)	69 (41.6%)	64 (38.6%)	
Age (years)	≤55	58 (17.4%)	30 (18.1%)	28 (16.9%)	0.879
	56–60	42 (12.7%)	20 (12.0%)	22 (13.2%)	
	61–65	65 (19.6%)	32 (19.3%)	33 (19.9%)	
	66–70	74 (22.3%)	34 (20.5%)	40 (24.1%)	
	> 70	93 (28.0%)	50 (30.1%)	43 (25.9%)	
Location	Rectum	171 (51.5%)	88 (53.0%)	83 (50.0%)	0.583
	Colon	161 (48.5%)	78 (47.0%)	83 (50.0%)	
Size (cm)	≤5.0	183 (55.1%)	92 (55.4%)	91 (54.8%)	0.833
	> 5.0	143 (44.0%)	72 (43.4%)	74 (44.6%)	
	Unknown	3 (0.9%)	2 (1.2%)	1 (0.6%)	
Differentiation	Well-moderate	309 (93.1%)	154 (92.8%)	155 (93.4%)	0.829
	Poor	23 (6.9%)	12 (7.2%)	11 (6.6%)	
T category	3	127 (38.3%)	62 (37.3%)	65 (39.2%)	0.735
	4	205 (61.7%)	104 (62.7%)	101 (60.8%)	
CEA^b^ (ng/mL)	< 5	175 (52.7%)	88 (53.0%)	87 (52.4%)	0.968
	≥5	118 (35.5%)	58 (34.9%)	60 (36.1%)	
	Unknown	39 (11.8%)	20 (12.1%)	19 (11.5%)	
Examined lymph nodes	< 12	84 (25.3%)	41 (24.7%)	43 (25.9%)	0.801
	≥12	248 (74.7%)	125 (75.3%)	123 (74.1%)	
Vessel carcinoma embolus	Negative	320 (96.4%)	160 (96.4%)	160 (96.4%)	1.000
	Positive	12 (3.6%)	6 (3.6%)	6 (3.6%)	
CCVD^c^	Negative	287 (86.4%)	147 (88.6%)	140 (84.3%)	0.262
	Positive	45 (13.6%)	19 (11.4%)	26 (15.7%)	
PLR^d^	≤130	176 (53.0%)	85 (51.2%)	91 (54.8%)	0.509
	> 130	156 (47.0%)	81 (48.8%)	75 (45.2%)	

^a^ P value of the Chi-square test

^b^ CEA: carcinoembryonic antigen

^c^ CCVD: cerebrovascular and cardiovascular diseases

^d^ PLR: platelet to lymphocyte ratio

**Fig. 5:**
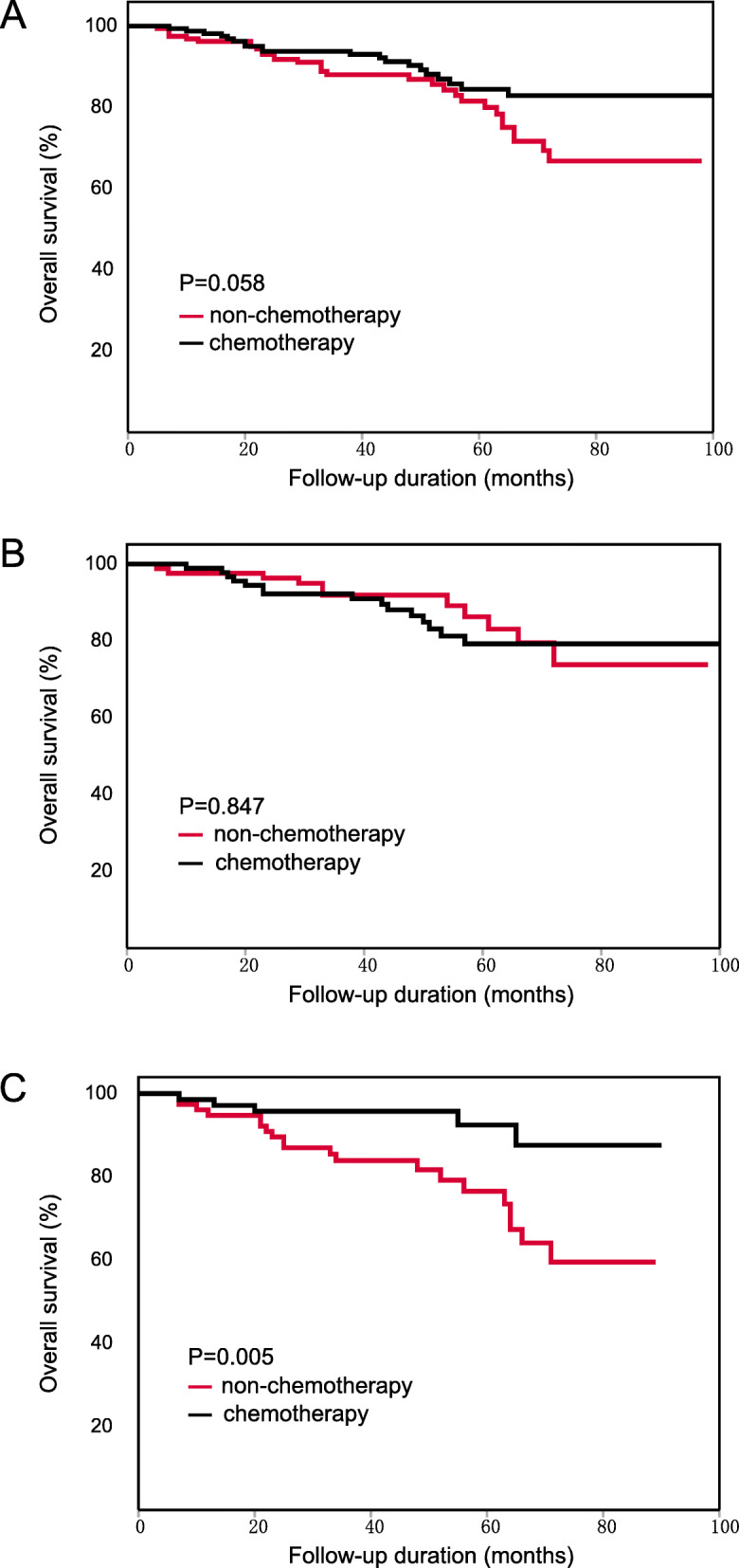
Kaplan-Meier OS curve of patients with stage II CRC after PSM. (**A**) The result of all patients. (**B**) The result of the low-PLR subgroup. (**C**) The result of the high-PLR subgroup

In contrast to the results before PSM, the chemotherapy patients did not have a longer OS than the non-chemotherapy patients (HR: 0.584, 95% CI: 0.333–1.025, Fig. 5A). The results of subgroup analysis showed that the chemotherapy patients with the following characteristics had a longer OS than the non-chemotherapy patients: male (HR: 0.487, 95% CI: 0.240–0.991), T4 category (HR: 0.501, 95% CI: 0.267–0.939), number of examined lymph nodes≥12 (HR: 0.404, 95% CI: 0.185–0.882) and PLR level > 130 (HR: 0.272, 95% CI: 0.102–0.726). The detailed results are shown in Table 7.

**Table 7:** OS analysis for patients after PSM

Characteristics		RMST^b^ (95%CI)		HR^c^ (95%CI)	P value^a^	Interaction p
		Non- Chemotherapy	Chemotherapy			
Total	–	53.3 (50.9–55.2)	56.0 (54.0–57.4)	0.584 (0.333–1.025)	0.058	–
Sex	Male	52.0 (48.1–54.6)	55.9 (53.2–57.6)	0.487 (0.240–0.991)	0.042	0.157
	Female	55.0 (50.7–57.2)	56.1 (52.8–58.0)	0.787 (0.310–2.003)	0.615	
Age (years)	≤55	53.8 (46.6–57.2)	56.6 (50.7–59.0)	0.536 (0.128–2.251)	0.386	0.574
	56–60	54.6 (45.6–58.2)	57.1 (48.8–59.2)	0.584 (0.097–3.499)	0.551	
	61–65	53.2 (45.7–57.0)	55.6 (49.8–58.3)	0.641 (0.171–2.403)	0.505	
	66–70	54.2 (47.2–57.3)	57.5 (52.6–59.1)	0.392 (0.098–1.576)	0.170	
	> 70	52.0 (46.9–55.2)	53.9 (49.1–56.9)	0.748 (0.305–1.830)	0.522	
Location	Rectum	52.4 (48.6–55.0)	54.6 (51.2–56.8)	0.725 (0.366–1.435)	0.353	0.283
	Colon	54.5 (50.6–56.9)	57.5 (54.9–58.9)	0.428 (0.158–1.163)	0.086	
Size (cm)	≤5.0	54.3 (50.8–56.6)	56.7 (54.0–58.1)	0.548 (0.236–1.269)	0.153	0.165
	> 5.0	52.6 (48.6–55.4)	55.1 (51.8–57.3)	0.672 (0.312–1.449)	0.307	
Differentiation	Well-moderate	53.5 (50.8–55.4)	56.1 (54.0–57.5)	0.570 (0.315–1.031)	0.059	0.702
	Poor	50.5 (36.8–56.9)	53.8 (39.9–58.2)	0.711 (0.117–4.339)	0.708	
T category	3	58.1 (54.7–59.3)	57.3 (54.2–58.9)	1.439 (0.342–6.045)	0.618	0.072
	4	50.9 (47.1–53.4)	55.2 (52.1–57.0)	0.501 (0.267–0.939)	0.027	
CEA^d^ (ng/mL)	< 5	55.2 (51.8–57.1)	57.3 (54.4–58.7)	0.557 (0.219–1.414)	0.211	0.790
	≥5	52.4 (46.8–55.7)	55.0 (51.0–57.4)	0.641 (0.265–1.552)	0.320	
Examined lymph nodes	< 12	50.7 (44.5–54.8)	51.7 (46.5–55.1)	0.898 (0.381–2.118)	0.806	0.322
	≥12	54.1 (51.4–56.0)	57.5 (55.5–58.7)	0.404 (0.185–0.882)	0.018	
PLR^e^	≤130	55.3 (51.8–57.4)	54.9 (52.0–56.8)	1.080 (0.495–2.355)	0.847	0.038
	> 130	51.3 (47.2–54.2)	57.6 (54.2–59.0)	0.272 (0.102–0.726)	0.005	

^a^ P value of the log-rank test

^b^ RMST: the restricted mean survival time

^c^ HR: Hazard Ratio, chemotherapy patients vs. non-chemotherapy patients

^d^ CEA: carcinoembryonic antigen

^e^ PLR: platelet to lymphocyte ratio

According to multivariate Cox survival analysis, PLR was still the only characteristic significantly associated with the effects of chemotherapy (interaction *p* = 0.038). In the low-PLR subgroup, the chemotherapy patients did not obtain OS benefits beyond those of the non-chemotherapy patients. (HR: 1.080, 95% CI: 0.495–2.355, Fig. 5B). In the high-PLR subgroup, the chemotherapy patients had a significantly longer OS than the non-chemotherapy patients (HR: 0.272, 95% CI: 0.102–0.726, Fig. 5C). The results after PSM further confirmed that PLR could predict the effects of chemotherapy in patients with stage II CRC.

### Cancer-specific survival analysis

**Fig. 6:**
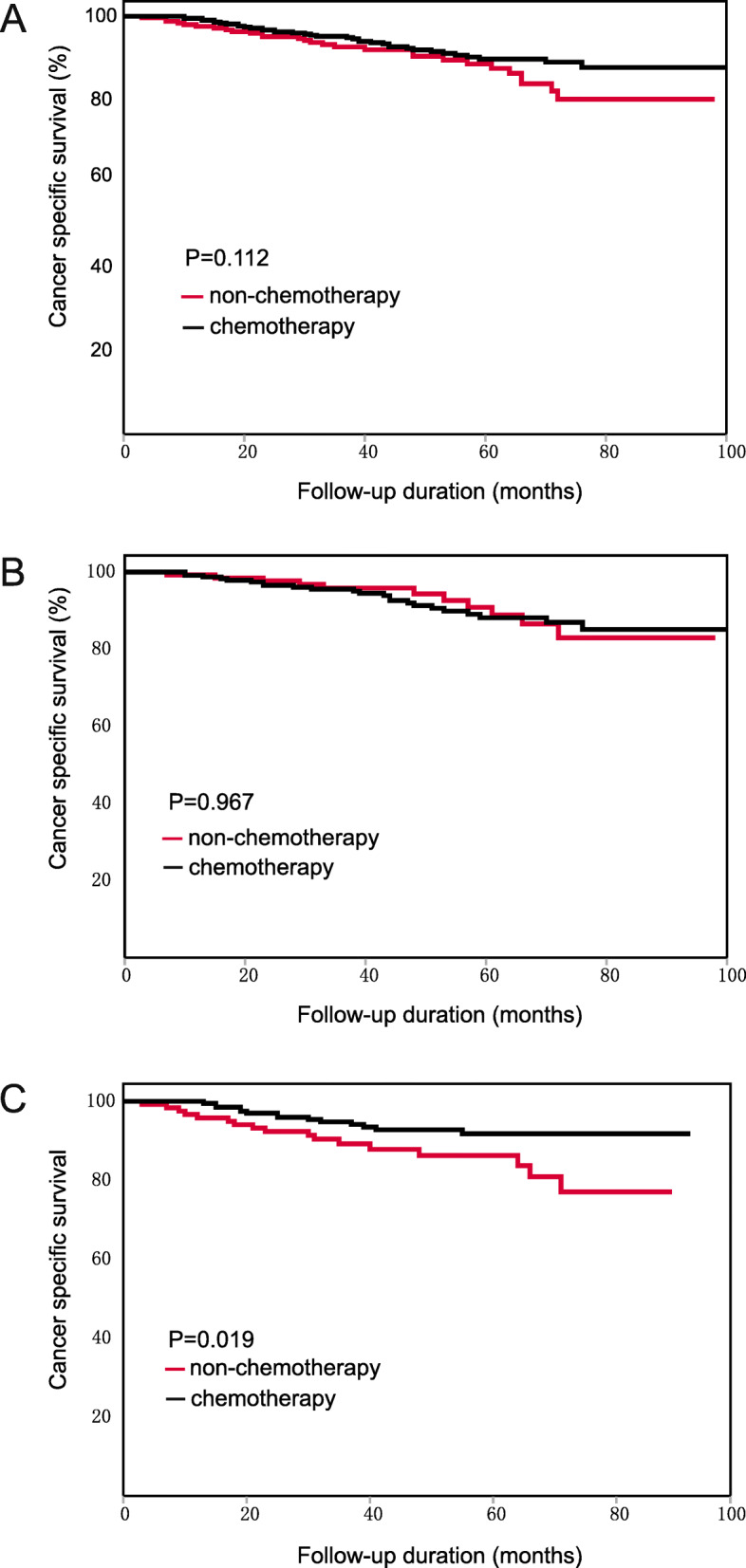
Kaplan-Meier CSS curve of patients with stage II CRC. (**A**) The result of all patients. (**B**) The result of the low-PLR subgroup. (**C**) The result of the high-PLR subgroup

For all patients, the chemotherapy patients did not obtain significant CSS benefits beyond those of the non-chemotherapy patients (HR: 0.673, 95%CI: 0.412–1.101, Fig. 6A). According to subgroup analysis, chemotherapy patients with the following characteristics had a longer CSS than the non-chemotherapy patients: 66–70 years old (HR: 0.172, 95% CI: 0.036–0.834) and PLR level > 130 (HR: 0.440, 95% CI: 0.217–0.893). The detailed results are shown in Table 8.

**Table 8:** CSS analysis for all patients

Characteristics		RMST^b^ (95%CI)		HR^c^ (95%CI)	P value^a^	Interaction p
		Non-chemotherapy	Chemotherapy			
Total	–	53.8 (51.7–55.3)	56.2 (50.1–57.1)	0.673 (0.412–1.101)	0.112	–
Sex	Male	53.2 (50.7–55.3)	55.9 (54.4–57.1)	0.598 (0.330–1.084)	0.086	0.393
	Female	54.5 (51.3–56.5)	56.8 (55.0–58.0)	0.882 (0.365–2.134)	0.781	
Age (years)	≤55	54.3 (47.9–57.4)	56.3 (54.2–57.7)	0.669 (0.218–2.053)	0.479	0.986
	56–60	55.4 (47.4–58.5)	56.8 (54.2–58.3)	0.656 (0.132–3.250)	0.602	
	61–65	53.3 (46.5–57.0)	55.7 (52.8–57.4)	0.720 (0.224–2.314)	0.519	
	66–70	53.2 (48.2–56.3)	57.3 (53.7–58.8)	0.172 (0.036–0.834)	0.013	
	> 70	53.6 (50.6–55.8)	54.6 (50.0–57.2)	1.240 (0.458–3.356)	0.671	
Location	Rectum	52.2 (49.2–54.5)	55.6 (53.8–56.9)	0.586 (0.311–1.105)	0.094	0.445
	Colon	55.2 (52.6–56.9)	56.9 (55.4–58.0)	0.775 (0.355–1.691)	0.521	
Size (cm)	≤5.0	54.0 (51.3–55.9)	56.1 (54.6–57.2)	0.737 (0.389–1.396)	0.347	0.154
	> 5.0	53.7 (50.4–55.8)	56.3 (54.4–57.5)	0.638 (0.289–1.408)	0.262	
Differentiation	Well-moderate	53.9 (51.9–55.4)	56.3 (55.1–57.2)	0.685 (0.406–1.157)	0.154	0.548
	Poor	52.0 (43.1–56.2)	54.7 (47.5–58.1)	0.625 (0.139–2.805)	0.535	
T category	3	56.4 (54.1–58.0)	57.6 (55.4–58.7)	0.797 (0.307–2.069)	0.640	0.377
	4	51.8 (48.7–54.0)	55.6 (54.2–56.7)	0.579 (0.326–1.030)	0.059	
CEA^d^ (ng/mL)	< 5	54.6 (51.9–56.6)	56.8 (55.3–57.8)	0.774 (0.376–1.596)	0.487	0.554
	≥5	53.5 (50.2–55.7)	55.1 (52.6–56.8)	0.682 (0.300–1.549)	0.357	
Examined lymph nodes	< 12	52.0 (47.8–54.9)	53.6 (50.4–55.7)	0.906 (0.407–2.018)	0.810	0.517
	≥12	54.4 (52.2–56.1)	57.1 (55.9–57.9)	0.590 (0.316–1.102)	0.094	
PLR^e^	≤130	56.0 (53.7–57.6)	56.0 (54.4–57.1)	1.016 (0.494–2.087)	0.967	0.116
	> 130	51.3 (48.2–53.8)	56.5 (54.9–57.8)	0.440 (0.217–0.893)	0.019	

^a^ P value of the log-rank test

^b^ RMST: the restricted mean survival time

^c^ HR: Hazard Ratio, chemotherapy patients vs. non-chemotherapy patients

^d^ CEA: carcinoembryonic antigen

^e^ PLR: platelet to lymphocyte ratio

The results of multivariate Cox survival analysis showed that PLR was not significantly associated with the effects of chemotherapy (interaction *p* = 0.116). However, CSS benefits form chemotherapy between the low-PLR and high-PLR subgroup markedly differed. In the low-PLR subgroup, the chemotherapy patients did not have a longer CSS than the non-chemotherapy patients (HR: 1.016, 95% CI: 0.494–2.087, Fig. 6B). In the high-PLR subgroup, the chemotherapy patients had a significantly longer CSS than the non-chemotherapy patients (HR: 0.440, 95% CI: 0.217–0.893, Fig. 6C). The results indicated that PLR was still associated with the effects of chemotherapy measured by CSS.

**Fig. 7:**
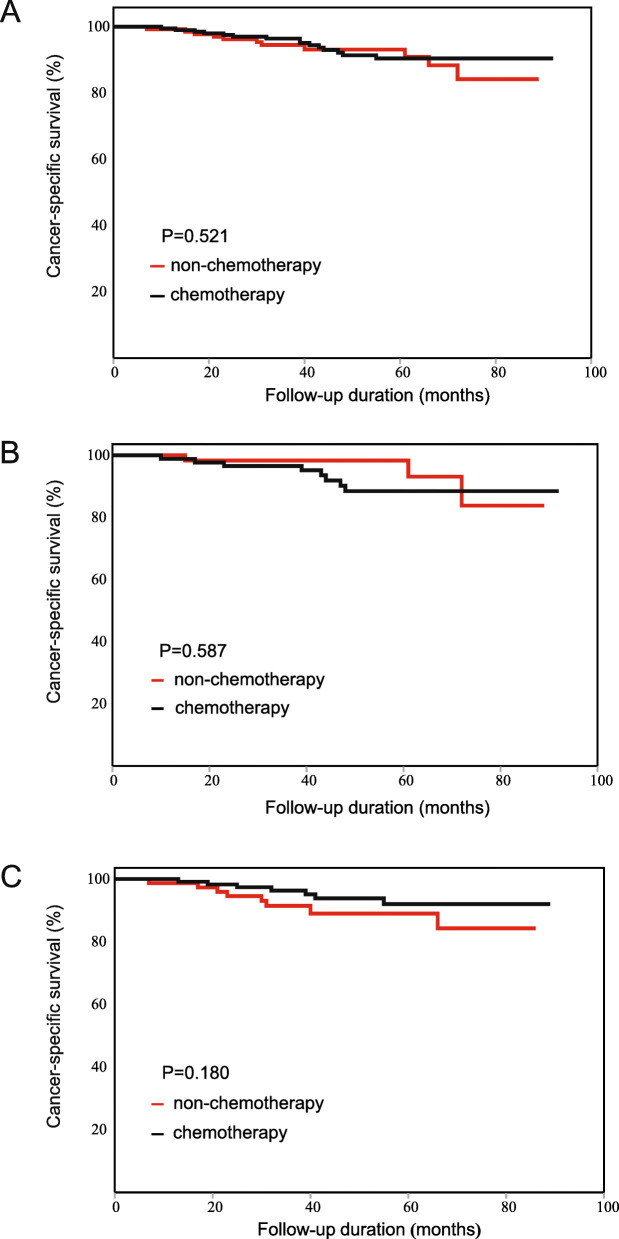
Kaplan-Meier CSS curve of patients with stage II colon cancer. (**A**) The result of all patients. (**B**) The result of the low-PLR subgroup. (**C**) The result of the high-PLR subgroup

**Fig. 8:**
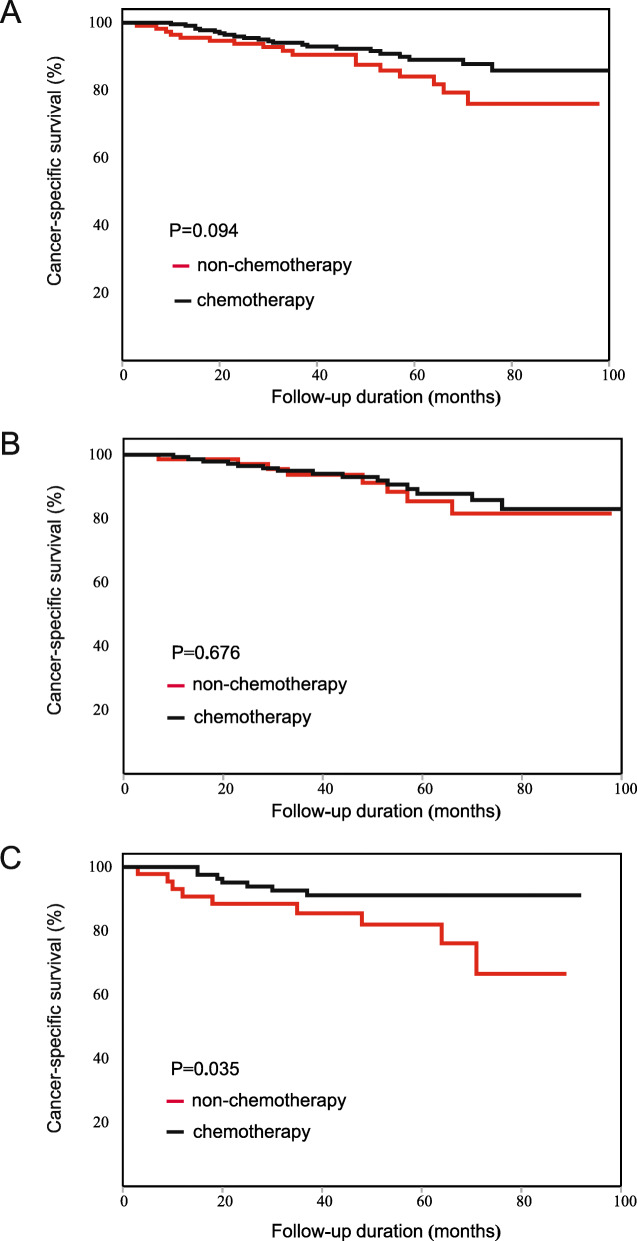
Kaplan-Meier CSS curve of patients with stage II rectal cancer. (**A**) The result of all patients. (**B**) The result of the low-PLR subgroup. (**C**) The result of the high-PLR subgroup

According to survival analyses in the colon cancer subgroup and the rectal cancer subgroup, PLR was associated with the effects of chemotherapy in both subgroups. The results were in accordance with the results for all patients. For colon cancer: the chemotherapy patients did not have a longer CSS than the non-chemotherapy patients (HR: 0.775, 95%CI: 0.355–1.691, Fig. 7A). In the low-PLR subgroup, the chemotherapy patients did not have a longer CSS (HR: 1.443, 95%CI: 0.381–5.464, Fig. 7B). In the high-PLR subgroup, the chemotherapy patients had a tendency toward longer CSS than the non-chemotherapy patients, although the results were not statistically significant (HR: 0.506, 95% CI: 0.183–1.397, Fig. 7C). For rectal cancer: the chemotherapy patients did not have a longer CSS than the non-chemotherapy patients (HR: 0.586, 95%CI: 0.311–1.105, Fig. 8A). In the low-PLR subgroup, the chemotherapy patients did not have a longer CSS (HR: 0.833, 95%CI: 0.352–1.967, Fig. 8B). In the high-PLR subgroup, the chemotherapy patients had a significantly longer CSS (HR: 0.360, 95% CI: 0.134–0.969, Fig. 8C). The detailed results are shown in Tables 9 and 10.

**Table 9:** CSS analysis for patients with colon cancer

Characteristics		RMST^b^ (95%CI)		HR^c^ (95%CI)	P value^a^	Interaction p
		Non-chemotherapy	Chemotherapy			
Total	–	53.8 (51.7–55.3)	56.2 (50.1–57.1)	0.775 (0.355–1.691)	0.521	–
Sex	Male	53.2 (50.7–55.3)	55.9 (54.4–57.1)	0.748 (0.259–2.164)	0.591	0.805
	Female	54.5 (51.3–56.5)	56.8 (55.0–58.0)	0.819 (0.260–2.585)	0.733	
Age (years)	≤55	54.3 (47.9–57.4)	56.3 (54.2–57.7)	0.384 (0.074–1.999)	0.237	0.371
	56–60	55.4 (47.4–58.5)	56.8 (54.2–58.3)	2.720 (0.001–16.94)	0.510	
	61–65	53.3 (46.5–57.0)	55.7 (52.8–57.4)	1.375 (0.159–11.91)	0.772	
	66–70	53.2 (48.2–56.3)	57.3 (53.7–58.8)	0.015 (0.001–19.84)	0.023	
	> 70	53.6 (50.6–55.8)	54.6 (50.0–57.2)	2.409 (0.486–11.96)	0.266	
Size (cm)	≤5.0	54.0 (51.3–55.9)	56.1 (54.6–57.2)	1.089 (0.364–3.259)	0.878	0.294
	> 5.0	53.7 (50.4–55.8)	56.3 (54.4–57.5)	0.554 (0.178–1.721)	0.300	
Differentiation	Well-moderate	53.9 (51.9–55.4)	56.3 (55.1–57.2)	0.777 (0.332–1.822)	0.561	0.669
	Poor	52.0 (43.1–56.2)	54.7 (47.5–58.1)	0.894 (0.125–6.367)	0.911	
T category	3	56.4 (54.1–58.0)	57.6 (55.4–58.7)	0.827 (0.137–4.990)	0.836	0.616
	4	51.8 (48.7–54.0)	55.6 (54.2–56.7)	0.717 (0.302–1.705)	0.450	
CEA^d^ (ng/mL)	< 5	54.6 (51.9–56.6)	56.8 (55.3–57.8)	0.721 (0.228–2.280)	0.576	0.663
	≥5	53.5 (50.2–55.7)	55.1 (52.6–56.8)	1.047 (0.294–3.726)	0.944	
Examined lymph nodes	< 12	52.0 (47.8–54.9)	53.6 (50.4–55.7)	0.857 (0.245–2.996)	0.809	0.962
	≥12	54.4 (52.2–56.1)	57.1 (55.9–57.9)	0.860 (0.311–2.376)	0.771	
PLR^e^	≤130	56.0 (53.7–57.6)	56.0 (54.4–57.1)	1.443 (0.381–5.464)	0.587	0.245
	> 130	51.3 (48.2–53.8)	56.5 (54.9–57.8)	0.506 (0.183–1.397)	0.180	

^a^ P value of the log-rank test

^b^ RMST: the restricted mean survival time

^c^ HR: Hazard Ratio, chemotherapy patients vs. non-chemotherapy patients

^d^ CEA: carcinoembryonic antigen

^e^ PLR: platelet to lymphocyte ratio

**Table 10:** CSS analysis for patients with rectal cancer

Characteristics		RMST^b^ (95%CI)		HR^c^ (95%CI)	P value^a^	Interaction p
		Non-chemotherapy	Chemotherapy			
Total	–	53.8 (51.7–55.3)	56.2 (50.1–57.1)	0.586 (0.311–1.105)	0.094	–
Sex	Male	53.2 (50.7–55.3)	55.9 (54.4–57.1)	0.515 (0.251–1.056)	0.065	0.488
	Female	54.5 (51.3–56.5)	56.8 (55.0–58.0)	0.989 (0.245–3.983)	0.987	
Age (years)	≤55	54.3 (47.9–57.4)	56.3 (54.2–57.7)	0.965 (0.205–4.548)	0.964	0.630
	56–60	55.4 (47.4–58.5)	56.8 (54.2–58.3)	0.463 (0.085–2.527)	0.362	
	61–65	53.3 (46.5–57.0)	55.7 (52.8–57.4)	0.466 (0.109–1.985)	0.290	
	66–70	53.2 (48.2–56.3)	57.3 (53.7–58.8)	0.329 (0.046–2.369)	0.246	
	> 70	53.6 (50.6–55.8)	54.6 (50.0–57.2)	0.833 (0.221–3.143)	0.787	
Size (cm)	≤5.0	54.0 (51.3–55.9)	56.1 (54.6–57.2)	0.575 (0.261–1.269)	0.165	0.342
	> 5.0	53.7 (50.4–55.8)	56.3 (54.4–57.5)	0.711 (0.232–2.177)	0.549	
Differentiation	Well-moderate	53.9 (51.9–55.4)	56.3 (55.1–57.2)	0.608 (0.313–1.180)	0.137	0.900
	Poor	52.0 (43.1–56.2)	54.7 (47.5–58.1)	0.372 (0.033–4.146)	0.403	
T category	3	56.4 (54.1–58.0)	57.6 (55.4–58.7)	0.792 (0.255–2.456)	0.685	0.376
	4	51.8 (48.7–54.0)	55.6 (54.2–56.7)	0.465 (0.215–1.003)	0.046	
CEA^d^ (ng/mL)	< 5	54.6 (51.9–56.6)	56.8 (55.3–57.8)	0.792 (0.312–2.013)	0.624	0.681
	≥5	53.5 (50.2–55.7)	55.1 (52.6–56.8)	0.457 (0.153–1.366)	0.151	
Examined lymph nodes	< 12	52.0 (47.8–54.9)	53.6 (50.4–55.7)	0.950 (0.324–2.786)	0.926	0.249
	≥12	54.4 (52.2–56.1)	57.1 (55.9–57.9)	0.444 (0.199–0.990)	0.041	
PLR^e^	≤130	56.0 (53.7–57.6)	56.0 (54.4–57.1)	0.833 (0.352–1.967)	0.676	0.202
	> 130	51.3 (48.2–53.8)	56.5 (54.9–57.8)	0.360 (0.134–0.969)	0.035	

^a^ P value of the log-rank test

^b^ RMST: the restricted mean survival time

^c^ HR: Hazard Ratio, chemotherapy patients vs. non-chemotherapy patients

^d^ CEA: carcinoembryonic antigen

^e^ PLR: platelet to lymphocyte ratio

### Cancer-specific survival analysis after PSM

**Fig. 9:**
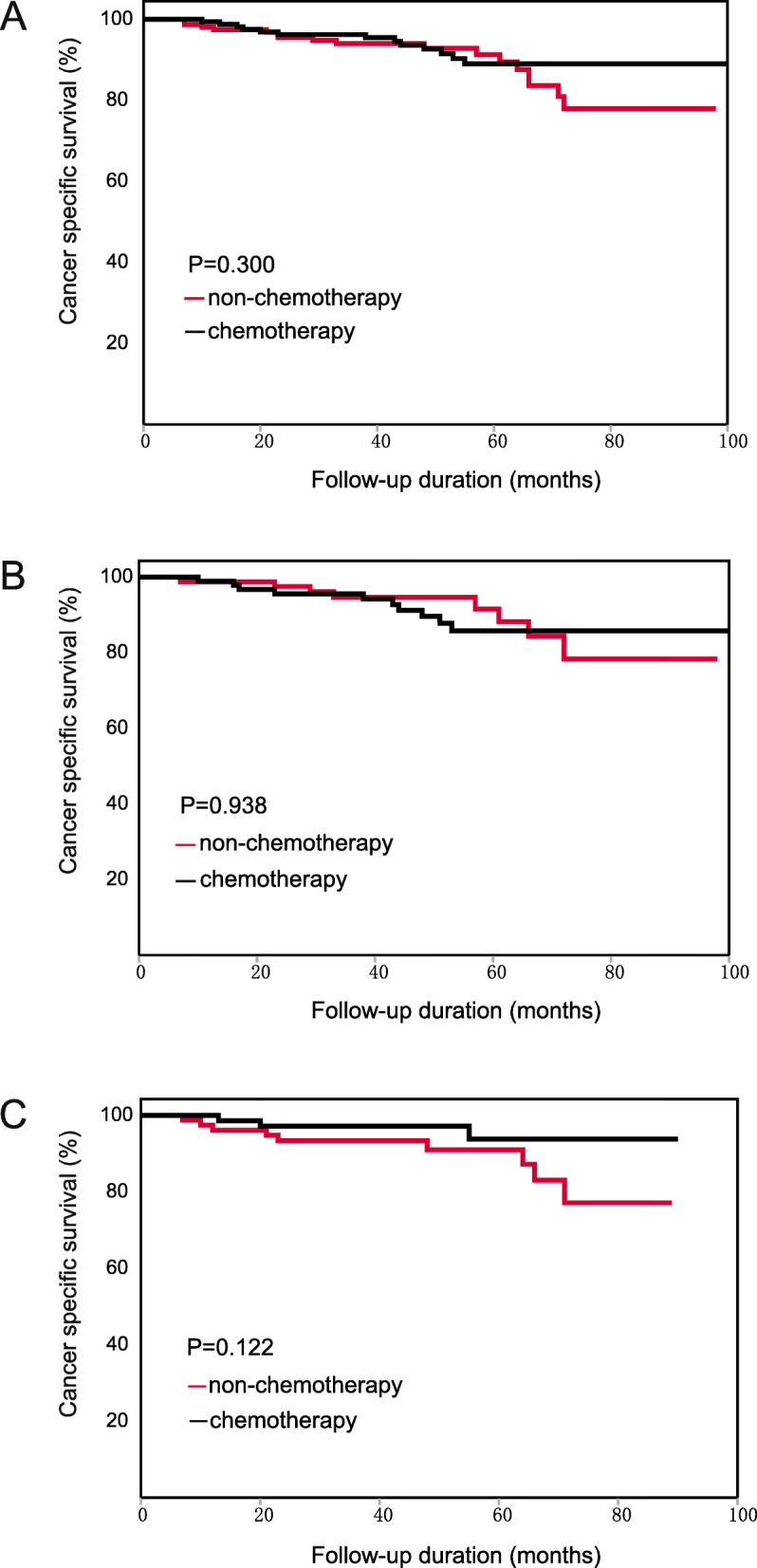
Kaplan-Meier CSS curve of patients with stage II CRC after PSM. (**A**) The result of all patients. (**B**) The result of the low-PLR subgroup. (**C**) The result of the high-PLR subgroup

For the 332 matched patients, the chemotherapy patients did not have a longer CSS than the non-chemotherapy patients (HR: 0.684, 95%CI: 0.332–1.409, Fig. 9A). According to the results of subgroup analysis, no survival difference between the chemotherapy and non-chemotherapy patients was found in any subgroup. The results indicated that no characteristics were associated with the effects of chemotherapy. The detailed results are shown in Table 11.

**Table 11:** CSS analysis for patients after PSM

Characteristics		RMST^b^ (95%CI)		HR^c^ (95%CI)	P value^a^	Interaction p
		Non-chemotherapy	Chemotherapy			
Total	–	53.3 (51.0–55.1)	56.0 (53.8–57.5)	0.684 (0.332–1.409)	0.300	–
Sex	Male	52.0 (48.4–54.6)	55.9 (53.2–57.7)	0.420 (0.169–1.042)	0.053	0.026
	Female	55.0 (51.1–57.3)	56.1 (52.2–57.9)	1.926 (0.478–7.762)	0.349	
Age (years)	≤55	53.8 (46.5–57.4)	56.6 (50.4–58.9)	0.435 (0.079–2.388)	0.324	0.196
	56–60	54.6 (45.3–58.2)	57.1 (49.9–59.2)	0.458 (0.041–5.054)	0.513	
	61–65	53.2 (46.3–57.0)	55.6 (48.5–58.1)	0.789 (0.158–3.949)	0.772	
	66–70	54.2 (48.5–57.3)	57.5 (52.7–59.1)	0.182 (0.020–1.645)	0.086	
	> 70	52.0 (47.2–55.3)	53.9 (48.5–56.5)	1.660 (0.468–5.887)	0.427	
Location	Rectum	52.4 (48.7–54.9)	54.6 (51.3–56.8)	0.738 (0.297–1.836)	0.511	0.797
	Colon	54.5 (50.3–56.8)	57.5 (54.8–58.8)	0.634 (0.192–2.087)	0.449	
Size (cm)	≤5.0	54.3 (50.6–56.5)	56.7 (54.0–58.2)	0.846 (0.295–2.425)	0.756	0.116
	> 5.0	52.6 (48.8–55.5)	55.1 (51.6–57.3)	0.641 (0.228–1.802)	0.395	
Differentiation	Well-moderate	53.5 (50.8–55.5)	56.1 (54.2–57.5)	0.703 (0.329–1.504)	0.361	0.864
	Poor	50.5 (36.0–56.7)	53.8 (38.2–58.6)	0.574 (0.050–6.560)	0.651	
T category	3	58.1 (54.1–59.4)	57.3 (54.3–58.9)	3.306 (0.367–29.738)	0.258	0.042
	4	50.9 (47.3–53.6)	55.2 (52.3–57.0)	0.517 (0.228–1.172)	0.107	
CEA^d^ (ng/mL)	< 5	55.2 (51.9–57.2)	57.3 (54.3–58.7)	0.785 (0.239–2.573)	0.688	0.897
	≥5	52.4 (47.5–55.8)	55.0 (51.4–57.2)	0.667 (0.203–2.195)	0.502	
Examined lymph nodes	< 12	50.7 (43.8–54.8)	51.7 (45.5–55.2)	1.620 (0.487–5.393)	0.427	0.107
	≥12	54.1 (51.1–56.0)	57.5 (55.6–58.7)	0.359 (0.128–1.007)	0.042	
PLR^e^	≤130	55.3 (51.8–57.4)	54.9 (51.9–56.8)	0.963 (0.379–2.449)	0.938	0.231
	> 130	51.3 (47.4–54.1)	57.6 (54.7–58.9)	0.372 (0.100–1.374)	0.122	

^a^ P value of the log-rank test

^b^ RMST: the restricted mean survival time

^c^ HR: Hazard Ratio, chemotherapy patients vs. non-chemotherapy patients

^d^ CEA: carcinoembryonic antigen

^e^ PLR: platelet to lymphocyte ratio

In multivariate Cox survival analysis, PLR was not significantly associated with the effects of chemotherapy (interaction *p* = 0.231). In the low-PLR subgroup, the chemotherapy patients did not have a longer CSS than the non-chemotherapy patients (HR: 0.963, 95%CI: 0.379–2.449, Fig. 9B). In the high-PLR subgroup, the chemotherapy patients had a tendency toward longer CSS than the non-chemotherapy patients, although the results were not statistically significant (HR: 0.372, 95% CI: 0.100–1.374, Fig. 9C).

## Discussion

Guidelines suggest patients with high-risk stage II CRC should receive adjuvant chemotherapy [8–12]. The NCCN guidelines define patients meeting the following criteria as the high-risk population: T4 depth of invasion, peritumoral lymphatic/venous invasion, histologic grade of 3 or greater, R1-R2 margin status, bowel obstruction or perforation or fewer than 12 nodes retrieved. Furthermore, patients are also defined as high-risk by the ASCO guidelines if they have elevated carcinoembryonic antigen (CEA) (CEA > 5 ng/ml). Although these high-risk characteristics, as determined by the OS benefits, can distinguish the population with poorer prognosis from all patients with stage II CRC, they cannot predict additional survival benefits from chemotherapy in this population. Given the lack of statistically significance differences in survival between the chemotherapy and non-chemotherapy population and the side effects that chemotherapy may cause [35, 36], chemotherapy may do more harm than good for certain patients with stage II CRC. Given this background, novel biomarkers are needed to distinguish the population in which chemotherapy will be effective among patients with stage II CRC.

Substantial evidence indicates that the progression of a tumor depends on not only the tumor itself, but also the inflammatory response of the host [37]. The inflammatory response has been demonstrated to lead to impaired immune function and an anti-tumor immune response of the host. This response has been widely accepted as an important stage-independent indicator, particularly in renal, gastro–esophageal and colorectal cancers. In fact, inflammatory cells, such as neutrophils, lymphocytes, platelets and monocytes, have been used in prognostic prediction for CRC patients [17, 19, 21, 38]. Furthermore, some studies have demonstrated an interaction between inflammation and the response or resistance of chemotherapy [22–26, 39]. The results of these studies indicated that predicting the effects of chemotherapy with inflammatory biomarkers was feasible.

Many studies have been performed to explore the clinical application of PLR, an important inflammatory marker. Several studies have found that elevated PLR was an indicator of poor prognosis in patients with CRC [19, 40]. However, the clinical application of PLR in patients with stage II CRC remains controversial. Ozawa et al. have indicated that PLR could be used as a prognostic marker in patients with stage II CRC who have undergone curative surgery but not adjuvant chemotherapy [41]. Some studies have shown that elevated PLR was significantly associated with poor survival in both stage II and III CRC [42, 43], whereas You et al. have reported that PLR was associated with survival outcomes in stage III CRC but not in stage II CRC [44]. Notably, most studies on PLR’s clinical application have focused on prognosis but not the effects of chemotherapy. Our study aimed at providing chemotherapy guidance is substantially different from these studies.

To our knowledge, our study is the first to predict the effect of chemotherapy in patients with stage II CRC. We found that PLR was the only characteristic associated with the effects of chemotherapy among all characteristics examined, including other inflammatory markers. Patients with elevated PLR obtained a significant survival benefit from chemotherapy, whereas patients with low PLR did not. PLR was able to predict the effects of chemotherapy and to distinguish the population in which chemotherapy is effective among patients with stage II CRC. However, some of our results differed from the guidelines and previous studies. According to the guidelines, patients with poor pathological differentiation or inadequate nodal resection are recommended to receive chemotherapy, while this population did not obtain a survival benefit from chemotherapy in our study. The possible reasons for the contradictory result may be as follows: First, selection bias may have resulted from the retrospective and single-center nature of our study. Second, the sample sizes of the subgroups may have been insufficient. Third, chemotherapy is not significantly effective in patients with poor pathological differentiation and inadequate nodal resection, although this population is recommended to receive chemotherapy. Furthermore, PLR was not significantly associated with the effects of chemotherapy in CSS analysis. The possible reasons may be as follows: First, the relatively small number of events, owing the insufficient sample size and good prognosis in patients in our study. Second, the intervention of other factors intricated in survival. For example, among all patients with elevated PLR, cardio-vascular complications may be more common in the chemotherapy group than non-chemotherapy group. There are also some other limitations in our study. First, the inability to analyze the role of bowel obstruction and perforation. Inflammatory-related indicators, such as neutrophil, platelet, lymphocyte counts and albumin levels, would largely deviate from the general level in a setting of bowel obstruction or perforation, therefore, patients with bowel obstruction or perforation were excluded from our analysis. Secondly, because of the relatively smaller number of patients who received single-agent therapy, we compared only the survival benefits between chemotherapy and non-chemotherapy patients, further comparison among different chemotherapy regimens was not performed. Third, as an important factor of chemotherapy decision in stage II CRC, we did not include microsatellite instability (MSI) in our study due to the lack of information. Last, PSM was performed to balance the covariates between the chemotherapy and non-chemotherapy group and to minimize the bias. However, as a retrospective study, the bias could not be completely eliminated. We will focus on these limitations mentioned above in our following prospective research.

To date, many studies have been conducted to explore the roles of lymphocytes and platelets in tumor progression. Platelets promote angiogenesis, adhesion, and invasion by secreting angiogenic and tumor growth factors, such as transforming growth factor-beta (TGF β) and vascular epidermal growth factor (VEGF), in a tumor environment [45–47]. Platelets have also been demonstrated to prevent the killing of tumor cells by natural killer cells [48]. Furthermore, platelets promote other immune cells, such as lymphocytes and neutrophils, to infiltrate into tumor tissues and trigger further inflammatory progress by releasing chemokines and cytokines [49]. Consequently, high levels of platelets partly reflect systemic inflammation and increased metastization of neoplastic cells [50, 51]. Lymphocytes, the main components of the immune defense against malignancy of the host, can induce cytotoxic cell death and inhibit tumor cell proliferation and migration [52, 53]. Therefore, low levels of lymphocyte partly reflect an impaired activation of adaptive immunity and poor nutritional status [54, 55]. The studies above did not directly elaborate on the mechanisms of lymphocytes and platelets in chemotherapy. However, they provided ideas for our research.

We suggest three possible explanations for the significant association between elevated PLR and effective chemotherapy treatment shown in our study. First, previous clinical studies have demonstrated that patients with CRC with elevated PLR may have a poorer prognosis and shorter postoperative survival time than other patients [19, 40–44]. Because high-risk patients usually require additional therapy, this aspect may partly explain why this group of patients obtain additional survival benefits from chemotherapy. Second, platelets have been demonstrated to be critical for maintaining tumor vascular generation and integrity [56]. Furthermore, high platelets may be associated with a rich network of tumor vessels and increased transport of chemotherapeutic agents into tumors, thus leading to effective chemotherapy treatment. In fact, improving vascular function has long been discussed as a possibility to improve the treatment effect of chemotherapy [57]. Third, myelosuppression, as a common side effect of chemotherapy, is an important factor for the continuation of chemotherapy in clinical practice. In chemotherapy for CRC, two frequently used agents, oxaliplatin and capecitabine, have been demonstrated to induce myelosuppression, including thrombocytopenia [58–61]. Patients with elevated PLR may have greater tolerance to chemotherapy than other patients, thus allowing them to receive longer and larger doses of chemotherapy. This aspect may explain the association between elevated PLR and effective chemotherapy treatment.

Our results indicated a positive association between elevated PLR and effective chemotherapy treatment in patients with stage II CRC. PLR showed potential as a practical, inexpensive, highly reliable and easily available marker to predict the effect of chemotherapy. However, the potential mechanisms and the specific predictive ability of PLR should be validated in further prospective, larger population and multi-center studies.

## Conclusions

In conclusion, our study indicated that PLR was significantly associated with the effects of chemotherapy in patients with stage II CRC. Patients with elevated PLR > 130 obtained substantial survival benefits from chemotherapy, whereas patients with low PLR ≤ 130 did not. PLR can be used as an effective inflammatory marker to predict the effects of chemotherapy and to distinguish the population in which chemotherapy is effective among patients with stage II CRC.

## Supplementary Information

**Additional file 1.** The clinicopathological characteristics of each patient before PSM.

**Additional file 2.** The clinicopathological characteristics of each patient after PSM.

## Data Availability

All data generated and analyzed during this study are included in this published article and its supplementary information files.

## Abbreviations

CRC: colorectal cancer
CEA: carcinoembryonic antigen
CCVD: cerebrovascular and cardiovascular diseases
PLR: platelet to lymphocyte ratio
NLR: neutrophil to lymphocyte ratio
LMR: lymphocyte to monocyte ratio
PNI: prognostic nutritional index
STEPP: Subpopulation Treatment Effect Pattern Plot
OS: overall survival
HR: Hazard Ratio
RMST: restricted mean survival time
PSM: Propensity Score Matching
TGF β: transforming growth factor-beta
VEGF: vascular epidermal growth factor
CI: Confidence interval
